# Droplet Digital PCR Provides Highly Sensitive and Accurate Opsin Gene SNP Detection From Wild Primate Fecal Samples

**DOI:** 10.1002/ece3.70996

**Published:** 2025-02-11

**Authors:** Arthur G. Fernandes, Saúl Cheves Hernández, Ronald López Navaro, Shoji Kawamura, Amanda D. Melin

**Affiliations:** ^1^ Department of Anthropology and Archaeology University of Calgary Calgary Canada; ^2^ Área de Conservación Guanacaste Liberia Costa Rica; ^3^ Department of Integrated Biosciences, Graduate School of Frontier Sciences The University of Tokyo Kashiwa Japan; ^4^ Department of Medical Genetics University of Calgary Calgary Canada; ^5^ Alberta Children's Hospital Research Institute, University of Calgary Calgary Canada

## Abstract

Evaluating field‐sourced samples with poor‐quality and low‐quantity DNA, like animal feces, presents significant challenges in the field of molecular biology. Nonetheless, recent innovations in PCR technology are promoted as effective tools to overcome many of these issues. Here, we evaluate the efficiency of droplet digital PCR (ddPCR) as a method for color vision assessment from feces of white‐faced capuchins ( *Cebus imitator* ) and report frequencies of alleles and genotypes in a wild population. The sex‐linked color vision polymorphism of monkeys in the Americas is driven by single nucleotide polymorphisms (SNPs) in opsin genes at up to three tuning sites. DNA was extracted from fecal samples collected from 211 wild capuchins (53.1% males) in Sector Santa Rosa, Costa Rica: 56 were evaluated with ddPCR, 24 with both ddPCR and Sanger sequencing, and 141 with Sanger sequencing (historical dataset). The same opsins and genotypes were derived for each monkey using Sanger and ddPCR; however, the latter method was far more sensitive and required far fewer samples to reach a definitive genotype. Overall, the most frequent phenotypes were red and green/red. The distribution of genotypes was: Females (*N* = 99): green/red (35.4%), red/red (33.3%), green/yellow (14.1%), yellow/red (12.1%), yellow/yellow (4.0%), and green/green (1.0%); Males (*N* = 112): red (60.7%), yellow (23.2%), and green (16.1%). Overall, ddPCR was a reliable method for evaluating color vision noninvasively in wild capuchins with the advantage of excellent sensitivity and high‐throughput. ddPCR is highly robust to PCR inhibitors and can be potentially used to identify other disease‐related SNP mutations noninvasively in wild animals.

## Graphical abstract

This study evaluates the use of droplet digital PCR (ddPCR) for color vision assessment in wild white‐faced capuchins from fecal DNA samples, demonstrating its superior sensitivity compared to traditional Sanger sequencing. In analyzing the sex‐linked color vision polymorphism, ddPCR effectively identified opsin genotypes and revealed common phenotypes, with red and green/red being the most frequent. This reliable, high‐throughput approach shows promise for broader applications in detecting SNP mutations in wildlife noninvasively.

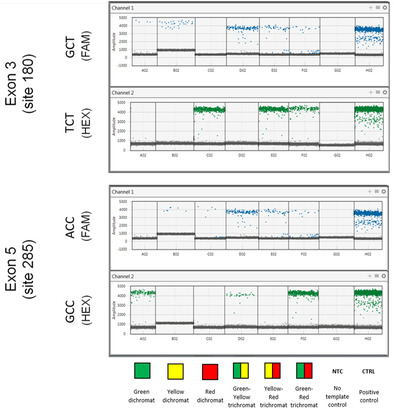

## 1 Introduction

Nucleic acid amplification and detection techniques are among the most valuable tools in molecular biology research. Such techniques have evolved over decades, from methods using polymerase chain reactions (PCR) followed by Sanger sequencing to various quantitative approaches (Schmitz et al. 2022; Zhu et al. 2020), which have fueled a multitude of discoveries and scientific advancements (Kumar et al. 2024; Schmitz et al. 2022). Despite these advances, working with field‐sourced samples with poor‐quality and low quantity DNA, such as animal feces, has consistently been an obstacle for researchers. This is due to a variety of confounding issues, such as compounds in the matrix that inhibit amplification, high risk of failure to detect some alleles (allelic dropout) due to low concentrations in the DNA of interest, and more (Verma et al. 2017). The latest advancement in PCR technology, droplet digital PCR, is often marketed as helping researchers overcome many of these issues. Given the importance and pervasive study of animal feces in biology and conservation ecology to understand host genetics, gut microbiomes, parasite dynamics, and much more, this could be an important breakthrough, but little research in this area has been conducted. Here, we contribute to this effort by comparing the performance of ddPCR to traditional PCR for detecting target SNPS of primate DNA from fecal samples.

PCR, first developed in the 1980s, involves a series of repeated heating and cooling cycles that allow for the selective replication of a target DNA sequence by denaturing double‐stranded molecules, annealing primers, and extension via polymerase (Zhu et al. 2020). The numerous applications of PCR include DNA sequencing, gene cloning, mutation analysis, genotyping, infectious disease diagnosis, forensic DNA profiling, and much more, being used as part of different molecular biology protocols (Valones et al. 2009). In the early 1990s, quantitative polymerase chain reaction (qPCR) was developed with the aim of quantifying the amount of target nucleic acid present in a sample. The fluorescence emitted by a fluorescent probe or dye is measured during each cycle, allowing for real‐time monitoring of the amplification process. This enables quantitative analysis of the amount of target sequences present in a sample. qPCR has numerous applications including gene expression analysis, microbial detection, viral load quantification, genotyping, and genetic disease diagnosis (Botes et al. 2013). It offers high sensitivity, specificity, and quantification capabilities; however, it is dependent on the standard curve generated and is also sensitive to inhibitors in nucleic acid samples, limiting its use in samples with low DNA concentrations and high impurities (Wang et al. 2022).

Droplet Digital PCR (ddPCR) is considered the third generation of PCR and involves the partitioning of a PCR reaction into thousands of droplets, each containing a small amount of the reaction mixture (Whale et al. 2016). PCR is then performed to determine the proportion of positive (with amplification) and negative (no amplification) partitions. This subdivision enables quantification to be performed using statistical models that are independent of a calibration curve and increases the precision of the quantification (Hindson et al. 2013). ddPCR has high sensitivity and can detect and quantify very low levels of target nucleic acids, even in the presence of high background noise or inhibitors (Kojabad et al. 2021). The digital nature of ddPCR, with individual droplet analysis, results in high precision and reproducibility, reducing variability and providing robust results. ddPCR has various applications in research and clinical settings, including rare mutation detection, copy number variation analysis, viral load quantification, gene expression analysis, and detection of circulating tumor DNA. Its ability to provide absolute quantification with high precision and sensitivity makes it a valuable tool in molecular biology and diagnostics. However, its application and suitability to different sample types are not yet well documented.

ddPCR may hold particular promise when applied to the amplification and analysis of DNA from field‐sourced samples where host DNA is of low quality and quantity and the matrix is high in amplification inhibitors that limit the success of standard PCRs, such as fecal samples. Previous studies have used ddPCR for pathogen detection such as viruses and bacteria, as well as for genetic testing for the detection and quantification of mutations, particularly in cancer research (Feng et al. 2023; Huang et al. 2021; Li et al. 2018; Olmedillas‐López et al. 2022). Here we test its ability to replace more traditional methods in SNP detection and identification by using fecal DNA collected from wild primates. We evaluate its efficacy for detecting SNPs in middle‐to‐long wavelength sensitive (M/LWS, OPN1LW) opsin genes underlying color vision status in platyrrhine primates. This is a well‐characterized system with an established genetic polymorphism present in the single‐copy opsin gene on the X‐chromosome.

The sex‐linked color vision polymorphism found among platyrrhines—species of monkeys in the Americas—is an area of long‐standing investigation. In most species, color vision variation is driven by SNPs at spectral tuning site 180 in exon 3 and sites 277 and 285 in exon 5 of the middle‐to‐long wavelength sensitive (M/LWS) opsin gene. This numbering system refers to the amino acid positions of bovine rhodopsin, which is standard practice for referencing spectral tuning sites in primate and other vertebrate M/LWS opsin sequences (Shichida and Matsuyama 2009). In white‐faced capuchins, there are three M/LWS alleles, each with a different amino acid composition across the three sites: the wavelength of maximal absorbance (λmax) at 560 nm (P560) (SYT), 545 nm (P545) (AFT), and 532 nm (P532) (AFA) (Kawamura et al. 2012; Kawamura 2018). The SYT, AFT, and AFA amino acid compositions of the M/LWS opsin gene confer sensitivity of the corresponding photopigments to red, yellow, and green light, respectively. All individuals also have an opsin gene coding for a short wavelength‐sensitive (SWS) opsin on an autosome. The M/LWS opsin gene is a classic case of sex‐linked heterozygosity, a condition where an individual possesses different alleles for a gene located on the sex chromosomes (X or Y chromosomes). Taken together, heterozygous females with two different M/LWS opsin alleles plus the SWS opsin gene are trichromatic, while (hemizygous) males and homozygous females have a single M/LWS opsin type and are dichromats. In total, six color vision phenotypes are possible. Although Sanger sequencing and melting curve analysis of PCR amplicons have been used to determine color vision genotypes, as with other applications, they can be time consuming, costly, and can have moderate to high failure rates, mainly associated with failing due to PCR inhibitors or failure to detect heterozygosity due to low DNA amounts.

The aims of the current study were two‐fold: (1) to evaluate the efficiency, sensitivity, and consistency of the droplet digital PCR (ddPCR) technique as a high‐throughput method for color vision assessment from the feces of white‐faced capuchins ( *Cebus imitator* ), relative to historical methods of PCR followed by Sanger sequencing; (2) To provide the most comprehensive report of the frequency of different alleles and genotypes in a wild population in Costa Rica to date and discuss its significance.

## 2 Methods

### 2.1 Study Population and Sample Collection

We ran ddPCR on fecal samples collected from 70 wild capuchins (50% females) from the Santa Rosa National Park, an area located in the tropical dry forest of northwest Costa Rica (10°50' N, 85°37' W). Fresh fecal samples were collected from individually known monkeys from the forest floor immediately following defecation by trained observers. We collected multiple samples from each primate. We collected the samples only when the observer was certain about the individuals' identification. We wore face masks and gloves to prevent any human contamination and placed fecal samples into sterile 15 mL falcon tubes with 5 mL of RNAlater. Samples were shipped to the University of Calgary, where we conducted DNA extraction and ddPCR analyses.

We additionally report Sanger sequencing results from 165 wild capuchins from the same population derived from our historical dataset, which were evaluated by PCR and Sanger sequencing on an ongoing basis from 2003 to 2016 at the University of Tokyo. A subset (24 monkeys) are also represented in the individuals studied with ddPCR.

### 2.2 DNA Extraction

DNA was extracted using either the NucleoSpin Tissue kit (MACHEREY‐NAGEL, France) or Qiagen DNeasy stool kit (QIAGEN, Germany) according to the manufacturer's instructions in two 50 μL elutions. DNA concentration was quantified by Qubit Flourometric Quantification (Life Technologies—Invitrogen, USA). All historical samples were extracted with the QIAGEN kits. For the newly collected samples, we started with the Qiagen kit but later switched to the NucleoSpin Tissue kit as it was more cost‐effective. Both kits performed similarly in terms of DNA yield and results.

### 2.3 Opsin Genotypes

We evaluated the genotype of the *M/LWS* gene. According to the SNPs identified in the spectral tuning site 180 in exon 3 and site 285 in exon 5, we identified alleles sensitive to red (P560), yellow (P545), or green (P532). Historically, all 3 sites (i.e., 180, 277, and 285) were analyzed, but due to complete linkage disequilibrium, the opsin genotype can be reliably assessed by examining 2 sites (180 in exon 3 and 285 in exon 5). As a way to help minimize allele dropout (i.e., one allele of a heterozygous locus fails to amplify, resulting in an erroneous interpretation of the genotype as homozygous) or false positive results, we assigned a genotype for each individual when at least two separate fecal samples showed identical results, regardless of the technique applied (ddPCR in the newly collected samples, and Sanger in the historical data).

### 2.4 ddPCR

ddPCR was performed on the 140 collected samples from 70 capuchin individuals. We designed four different Affinity Plus Probes, two targeting alleles of the exon 3 tuning site 180 (one for T and one for G of the TCT/GCT polymorphic codon) and two targeting alleles of the exon 5 tuning site 285 (A and G of the ACC/GCC polymorphic codon; Table 1). Together, the combination of SNPs in these two locations captures the three different opsin alleles present in the population (Hiramatsu et al. 2005; Hiwatashi et al. 2010). To first test their ability to target the specific mutation, we generated synthetic DNA for the opsin genes, including all the different genotypes of interest. Besides testing the probes performance, the synthetic DNA was also used for the assay optimization by testing different primers and probes concentrations as well as optimizing cloud separation using a temperature gradient.

**TABLE 1:** Primers and Probes used to diagnose SNPs via ddPCR on exon 3 and 5 of the Cebus OPN1LW opsin gene.

Exon/ Tuning site	Primer/Probe	Oligo Name	bp	Tm (°C)	%GC	Sequence
Exon 3 site 180	Primers	IDT_Cebus_EX3_F	17	62.2	58.8	GGCTGGTTGTCTGCAAG
		IDT_Cebus_EX3_R	19	62.1	52.6	CTGCTCCAACCAAAGATGG
	Probes	IDT_Cebus_EX3(180)_P‐G	10	65.7	70.0	TCTGG **GCT**GC
		IDT_Cebus_EX3(180)_P‐T	11	66.0	54.5	ATCTGG **TCT**GC
Exon 5 site 285	Primers	IDT_Cebus_EX5_F2	19	61.6	52.6	CATGGTGGTGGTGATGATC
		IDT_Cebus_EX5_R2	16	61.9	62.5	GAGGGTGGAAGGCGTA
	Probes	IDT_Cebus_EX5(285)_P‐ACC	11	65.0	54.5	A**GGT** GTAGGGT
		IDT_Cebus_EX5(285)_P‐GCC	11	64.7	63.6	A**GGC** GTAGGGT

*Note:* The codon for the amino acid at the tuning site is indicated in bold font. The polymorphic SNPs responsible for amino acid differences are indicated in blue font.

Two duplex ddPCR assays using probes targeting the variation at exon 3 site 180 and exon 5 site 285 were analyzed using fluorescein amidine (FAM) and hexachloro fluorescein (HEX) fluorophores to target different SNPs. For each reaction, the samples were mixed with a solution containing a supermix, primers, and probes and were transferred into cartridges placed in the droplets generator. The generated droplets were transferred into a 96‐Well Plate for PCR amplification. The droplets were then read in a Droplet reader (BIORAD) determining the proportion of positive (with amplification) and negative (no amplification) droplets. In each run of samples, a positive control created with synthetic DNA and a non‐template control were included. Responses with amplitudes around 1000 were considered negatives, and only those with amplitudes between 3000 and 5000 were considered positives. These thresholds were established based on the results of positive control samples created with synthetic DNA and negative controls included in each experimental run. The full protocol for ddPCR is available as Appendix S2.

### 2.5 Sanger Sequencing

To assess the relative performance of ddPCR, we compared parameters to a historical dataset of fecal samples analyzed for opsin genotype using Sanger sequencing. Previously (Hiramatsu et al. 2005; Hiwatashi et al. 2010; Melin et al. 2014, 2017), we analyzed 517 fecal samples from 165 primates using traditional Sanger sequencing protocols. Of these individuals, 24 monkeys were also included in the analysis. However, we used newly collected samples because the originally analyzed samples were not available for ddPCR analyses. An in‐depth description of the Sanger method is presented in Hiwatashi et al. (2010). In brief, the DNA was extracted using QIAGEN (QIAamp DNA Stool Mini Kit). We then ran a PCR to amplify exon 3 and two separate PCRs to amplify the two tuning sites on exon 5 (Hiramatsu et al. 2005; Hiwatashi et al. 2010; Melin et al. 2014, 2017). The PCR products were isolated via gel excision, solubilized, purified, and subjected to Sanger sequencing using sequencing primers (Table 2).

**TABLE 2:** PCR Primer Pairs Used to amplify the OPN1LW Gene Regions for Sanger sequencing in Capuchin Monkeys.

Region		Forward	Reverse
OPN1LW opsin	Exon 3	CTGATTCCTTGCTCTTGGCT	ACATTCCCTCTCCAAACACC
	Exon 5	TCCCTCTCTCATCCCCACTCA	TGGATGTACCTAGGGCTCACC

### 2.6 Data Analysis

We used Stata 14.0 (StataCorp, College Station, TX, USA) for statistical analyses. Frequency tables were used for descriptive analysis. The allele frequency from males and females was compared by Fisher's Exact Test. We evaluated the agreement between ddPCR and Sanger results by the kappa agreement coefficient. *p* ≤ 0.05 was considered statistically significant.

We assessed the frequencies of the three different alleles: S(Y)T, red; A(F)T, yellow; A(F)A, green for each of the two datasets, and then we assessed the frequencies of the total individuals genotyped.

## 3 Results

### 3.1 Efficiency, Sensitivity, and Consistency of the Droplet Digital PCR (ddPCR) Technique

#### 3.1.1 ddPCR Results

Following the validation and optimization, we performed ddPCR in 140 samples from 70 individuals (two samples per monkey; Appendix S1). All samples successfully amplified, no allelic dropout has occurred, and we were able to assign a genotype to each sample (100% success rate). There were no discrepancies between the two samples from the same individual. We were able to identify 6 different genotypes by examining the profiles of positive and negative droplets in each of the channels (green‐sensitive dichromat, yellow‐sensitive dichromat, red‐sensitive dichromat, green‐yellow‐sensitive trichromat, yellow‐red‐sensitive trichromat, green‐red‐sensitive trichromat), as well as the results expected for non‐template controls and positive synthetic DNA controls.

**FIGURE 1:**
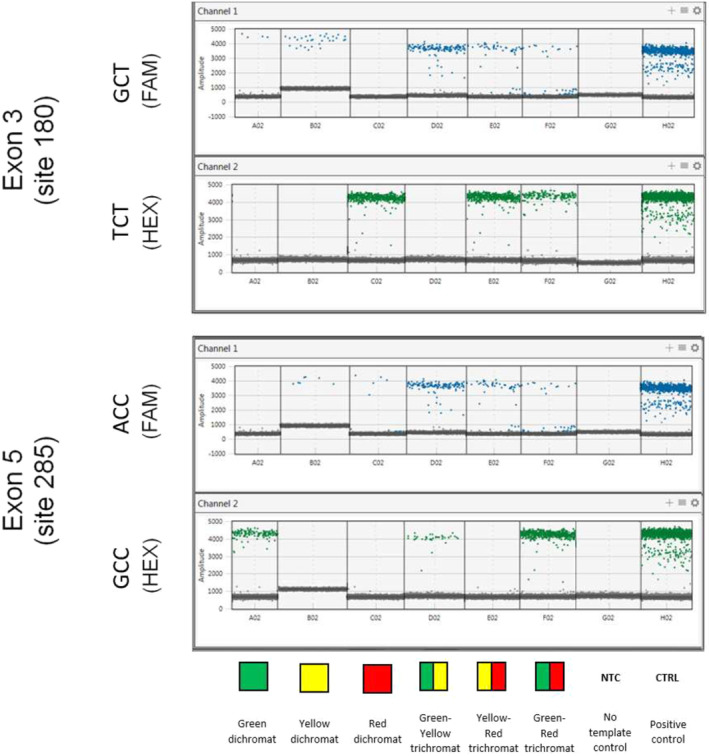
ddPCR results for different genotypes. GCT on site 180 + GCC on site 285 = Green dichromat; GCT on site 180 + ACC on site 285 = Yellow dichromat; TCT on site 180 + ACC on site 285 = Red dichromat; GCT on site 180 + ACC and GCC on site 285 = Green/Yellow trichromat; GCT and TCT on site 180 + ACC on site 285 = Yellow/Red trichromat; GCT and TCT on site 180 + ACC and GCC on site 285 = Green/Red trichromat.

Figure 1 shows examples of the six possible genotypes identified. Variability in FAM and HEX intensity is due to stochasticity in the fecal samples, such as the number of DNA copies containing the sequence of interest. We looked for inconsistencies that would indicate false positives, contamination, or allelic dropout, such as opsin heterozygosity in male samples or a homozygous and a heterozygous result present for different samples from the same female. Such scenarios were not detected in this study. Finally, given the very high sensitivity of ddPCR, any contamination would likely have been evident in the non‐template controls, and such samples would have been discarded to maintain the integrity of the results.

Each of the different genotypes was found in female capuchins (Table 3). The allele frequency for females was 39 Red alleles (55.71%), 17 Green alleles (24.29%), and 14 Yellow alleles (20.00%). The majority of females had a trichromat genotype (57.15%). The most common trichromatic genotype was Green/Red (34.29%) and the most common dichromatic genotype was Red/Red (31.43%; Table 3). For males, the allele frequency was 24 Red alleles (68.57%), 7 Green alleles (20.00%), and 4 Yellow alleles (11.43%). Among males, the Red genotype was the most frequent (68.57%) (Table 3). No significant differences were observed in allele frequency between males and females (*p* = 0.353).

**TABLE 3:** Frequency of opsin phenotypes in 70 wild capuchin monkeys ( *Cebus imitator* ) using ddPCR genotyping.

	Predicted Phenotype	Frequency
Males (*n* = 35) One X‐linked allele	Red	24 (68.57%)
	Green	7 (20.00%)
	Yellow	4 (11.43%)
Females (*n* = 35) Two X‐linked allele	Green/Red	12 (34.29%)
	Red/Red	11 (31.43%)
	Green/Yellow	4 (11.43%)
	Yellow/Red	4 (11.43%)
	Yellow/Yellow	3 (8.57%)
	Green/Green	1 (2.86%)

#### 3.1.2 Sanger Sequencing Results

Our historical dataset of individuals genotyped with Sanger sequencing counts 165 capuchins from the Santa Rosa National Park, with color vision assessed considering the criterion of two different samples retrieving the identical results. Out of the 517 tested samples, 330 successfully amplified (64% success rate) and we were able to assign a genotype to each individual. In this group, we also identified the 6 possible color vision genotypes. The allele frequency for females was 96 Red alleles (59.26%), 41 Green alleles (25.31%), and 25 Yellow alleles (15.43%). The majority of females had a trichromat genotype (62.96%). The most common trichromatic genotype was Green/Red (37.04%) and the most common dichromatic genotype was Red/Red 28 (34.57%; Table 4). For males, the allele frequency was 48 Red alleles (57.14%), 19 Green alleles (22.62%), and 17 Yellow alleles (20.24%). Among males, the Red genotype was the most frequent (57.14%) (Table 3). No significant differences were observed in allele frequency between males and females (*p* = 0.620).

**TABLE 4:** Frequency of phenotypes in 165 wild capuchin monkeys ( *Cebus imitator* ) using Sanger Sequencing genotyping from our historical dataset.

	Genotype	Frequency
Males (*n* = 84) One X‐linked allele	Red	48 (57.14%)
	Green	19 (22.62%)
	Yellow	17 (20.24%)
Females (*n* = 81) Two X‐linked allele	Green/Red	30 (37.04%)
	Red/Red	28 (34.57%)
	Green/Yellow	11 (13.58%)
	Yellow/Red	10 (12.35%)
	Yellow/Yellow	2 (2.47%)
	Green/Green	0 (0.00%)

#### 3.1.3 Sanger Sequencing vs. ddPCR

The subset of 24 individuals who were tested with both Sanger and ddPCR has shown identical results with 100% agreement and kappa = 1.00 (*p* < 0.001). Table 5 shows the frequencies of genotypes from the individuals tested with both methods.

**TABLE 5:** Frequency of genotyping among individuals tested with Sanger and ddPCR.

	Sanger	ddPCR
Red	11 (45.83%)	11 (45.83%)
Green/Red	6 (25.00%)	6 (25.00%)
Yellow	4 (16.67%)	4 (16.67%)
Yellow/Red	2 (8.33%)	2 (8.33%)
Green/Yellow	1 (4.17%)	1 (4.17%)
Green	0 (0.00%)	0 (0.00%)

The per sample success rate of opsin genotyping using ddPCR (100%) was significantly higher than the one for Sanger sequencing (63.8%). While 2 tested samples were enough to get a genotype using ddPCR, an average of 3.13 samples was needed per individual to successfully determine a genotype using Sanger Sequencing using the established criteria for determining an opsin genotype.

### 3.2 Frequency of Different Alleles and Genotypes in a Wild Population in Costa Rica

When combining the individuals evaluated by ddPCR (*n* = 70) and the individuals evaluated with Sanger sequencing (*n* = 165), we have a total of 211 individuals genotyped in our dataset (the 24 individuals measured with both methods were counted only once in the overall allele frequency analysis). The allele frequency for females was 113 red alleles (57.07%), 51 green alleles (25.76%), and 34 yellow alleles (17.17%). For males, the allele frequency was 68 red alleles (60.71%), 26 green alleles (23.21%), and 18 yellow alleles (16.08%). No significant differences were observed in allele frequency between males and females (*p* = 0.818). Also, no significant differences were observed in the allele frequencies when comparing the two datasets of ddPCR and Sanger methods (*p* = 0.996).

Each of the different genotypes is represented in females, with most of them showing a trichromat genotype (61.61%) with a higher frequency of green/red sensitivity (35.35%), while most of the dichromacy genotype (38.38%) showed red sensitivity (33.33%). Among males, the red genotype was the most frequent (60.71%) in this population. Table 6 shows the overall frequency of genotyping among males and females.

**TABLE 6:** Frequency of genotyping among all sampled males and females in the Santa Rosa National Park considering the entire dataset of individuals evaluated by ddPCR or Sanger.

	Genotype	Frequency
Males (*n* = 112) One X‐linked allele	Red	68 (60.71%)
	Green	26 (23.21%)
	Yellow	18 (16.08%)
Females (*n* = 99) Two X‐linked allele	Green/Red	35 (35.35%)
	Red/Red	33 (33.33%)
	Green/Yellow	14 (14.14%)
	Yellow/Red	12 (12.12%)
	Yellow/Yellow	4 (4.04%)
	Green/Green	1 (1.01%)

## 4 Discussion

### 4.1 Evaluation of ddPRC vs. Traditional PCR and Sequencing

We present evidence that ddPCR provides a robust and reproducible method to detect SNPs of interest using DNA extracted from fecal samples. The composition of SNPs from the individuals evaluated with ddPCR and Sanger was in complete agreement, validating the ddPCR method. The ddPCR method was fast, high‐throughput, and reliable, and thus has considerable promise for analyzing field‐sourced samples where host DNA is of low quality and quantity.

In comparison to traditional methods, ddPCR has several advantages. Our historical dataset of samples evaluated by Sanger included 517 samples from 165 individuals that went through Sanger sequencing. Out of the total samples, we were never able to amplify DNA from 182 samples, likely due to the inhibitors present in fecal samples. Additionally, 5 samples showed allelic dropout, which resulted in an overall success rate of 63.8% and an average of 3.13 samples needed per individual to get a successful genotype (i.e., two samples with reliable results). With ddPCR, we had a success rate of 100% as all the samples provided successful results, such that we needed only 2 samples tested to get a successful genotype. While it would have been ideal to analyze the exact same samples simultaneously using both methods, our comparison still strongly suggests that ddPCR is able to achieve results using fewer fecal samples without any notable errors. Moreover, ddPCR is a significantly faster method. Sanger sequencing takes multiple days for each of the steps, including PCR reaction, gel excision, and the Sanger sequencing itself. In comparison, ddPCR allows us to evaluate up to 48 samples (two duplex reactions for Exon 3 and Exon 5) in one single day, with the final results in a matter of hours.

While ddPCR has shown to be an excellent technique to evaluate SNP mutations in samples with low quality and quantity DNA, some limitations have to be noted. For example, ddPCR has been reported to show a higher false‐positive rate (Kojabad et al. 2021). In our study, we guard against this by assessing two samples from every individual when determining an individual's genotype, and no indication of false‐positive cases was observed. ddPCR can also be expensive due to the requirement of specialized equipment and reagents. However, when considering the time saving and successful result rate, the costs are not high overall, and cost assessment depends on the number of samples to be processed in a specific project.

Our results suggest ddPCR is effective when analyzing samples with low‐quality DNA. This raises the possibility of its use in a wide variety of contexts, including where samples are non‐invasive and of poorer quality. Potential other applications include evaluating pathogen‐based infections or the occurrence of specific disease‐related SNP mutations. The method has shown great sensitivity in SARS‐CoV‐2 detection, besides monitoring the disease course and treatment response of COVID‐19 patients (Ishak et al. 2021). In the study of cancer, ddPCR has emerged as a reliable and precise tool for detecting nucleic acid‐based markers derived from various sources such as cell‐free DNA, cell‐free RNA, circulating tumor cells, extracellular vesicles, or exosomes when isolated from whole blood, plasma, and serum, helping to anticipate tumor relapse or unveil intratumor heterogeneity and clonal evolution in response to treatment (Olmedillas‐López et al. 2022). Our data suggest its application in a wider range of clinical sciences, which might offer benefits to patients and be used in monitoring and diagnosis.

### 4.2 Color Vision Assessment of Wild Primates

Assessing the color vision status of wild animals has generated many diverse insights into sensory behavior, maintenance of genetic polymorphisms, and evolutionary biology of primates and other mammals (Hiramatsu et al. 2005; Jacobs and Bradley 2016; Kawamura et al. 2012; Melin et al. 2014; Surridge and Mundy 2002; Surridge et al. 2003). For example, previous studies of lemurs and monkeys in the Americas have used opsin genotyping to evaluate the impact of color vision on fruit intake, the use of other senses, such as smell, and food perception and assessment (Chaves et al. 2023; Hiramatsu et al. 2008, 2009; Jacobs et al. 2017; Melin et al. 2017, 2019; Veilleux and Bolnick 2009; Veilleux et al. 2022). Yet other studies have linked color vision and perception with sexual signaling and reproductive status (e.g., Bergman et al. 2009; Dubuc et al. 2009; Moreira, Watsa, et al. 2023; Moreira, Merrigan‐Johnson, et al. 2023).

The opsin results we report here for over 200 individual white‐faced capuchin monkeys provide the most comprehensive assessment of allelic diversity in a wild population of primates to date, and allow us to report a robust characterization of the distribution of opsin alleles. We find a significantly higher frequency of red alleles than expected if the system followed the Hardy–Weinberg equilibrium under which we would expect equal frequencies (33.33% each) of red, green, and yellow‐sensitive opsins. The skew favoring red‐sensitive alleles is consistent with studies examining smaller sample sizes and suggests that this allele is more favorable in this population, which could be driven by advantages to dichromats or trichromats for either ecological or social advantages (see Hiwatashi et al. 2010; Kawamura 2016 for hypotheses about evolutionary drivers). Determining the nature of these advantages is a goal for future work and should likely combine modeling and experimental approaches, as well as examining the lifetime fitness of individual primates with different color vision types (Fedigan et al. 2014; Hagen et al. 2023; Kawamura 2016).

We find that ddPCR is a reliable method for evaluating SNPs noninvasively from fecal samples that have typically been difficult to work with due to inhibitors or low‐quality DNA, with the added advantage of excellent sensitivity and very high‐throughput. We infer that ddPCR is highly robust to PCR inhibitors and can be potentially used to identify disease‐related SNP mutations noninvasively in clinical settings or in support of the study of genetic variation in wild animals. We additionally provide the most comprehensive report to date on the distribution of OPN1LW alleles in a wild primate population, using SNP detection by PCR. The OPN1LW gene is of longstanding interest to animal behaviorists, primatologists, and evolutionary biologists. Using ddPCR, similar studies could quickly generate OPN1LW profiles for many additional species using non‐invasively collected fecal samples.

## Author Contributions

**Arthur G. Fernandes:** conceptualization (equal), data curation (equal), formal analysis (equal), methodology (equal), writing – original draft (equal). **Saúl Cheves Hernández:** data curation (equal), investigation (equal), methodology (equal), writing – review and editing (equal). **Ronald López Navaro:** data curation (equal), investigation (equal), methodology (equal), writing – review and editing (equal). **Shoji Kawamura:** conceptualization (equal), data curation (equal), investigation (equal), writing – review and editing (equal). **Amanda D. Melin:** conceptualization (equal), funding acquisition (equal), project administration (equal), resources (equal), supervision (equal), writing – review and editing (equal).

## Conflicts of Interest

The authors declare no conflicts of interest.

## Supporting information


Appendix S1.

## Data Availability

The data supporting the results in the paper are publicly available as appendix (Appendix S1).
