# Supplementary material for: Droplet Digital PCR Provides Highly Sensitive and Accurate Opsin Gene SNP Detection From Wild Primate Fecal Samples
Source: Ecol Evol. 2025 Feb 11;15(2):e70996. doi: 10.1002/ece3.70996 (PMC11814474; doi:10.1002/ece3.70996)
Supplement: Supplementary file 1 — Appendix S1. [file ECE3-15-e70996-s001.docx]

APPENDIX 1

Appendix 1. Individual data on ddPCR testing and agreement with Sanger sequencing

| **Individual** | **Sex** | **Sample I** | | | | **Sample II** | | | | **DDPCR genotype** | **SANGER genotype** |
| --- | --- | --- | --- | --- | --- | --- | --- | --- | --- | --- | --- |
|  |  | *ID* | *EX3(180)* | *EX5(285)* | *Result* | *ID* | *EX3(180)* | *EX5(285)* | *Result* |  |  |
| 001 | M | 001A | **P_G** | **P_ACC** | YELLOW | 001B | **P_G** | **P_ACC** | YELLOW | YELLOW | YELLOW |
| 002 | F | 002A | **P_G** | **P_ACC + P_GCC** | GREEN/YELLOW | 002B | **P_G** | **P_ACC + P_GCC** | GREEN/YELLOW | GREEN/YELLOW | GREEN/YELLOW |
| 003 | F | 003A | **P_G + P_T** | **P_ACC + P_GCC** | GREEN/RED | 003B | **P_G + P_T** | **P_ACC + P_GCC** | GREEN/RED | GREEN/RED | GREEN/RED |
| 004 | F | 004A | **P_G** | **P_ACC + P_GCC** | GREEN/YELLOW | 004B | **P_G** | **P_ACC + P_GCC** | GREEN/YELLOW | GREEN/YELLOW | NA |
| 005 | F | 005A | **P_G + P_T** | **P_ACC + P_GCC** | GREEN/RED | 005B | **P_G + P_T** | **P_ACC + P_GCC** | GREEN/RED | GREEN/RED | GREEN/RED |
| 006 | F | 006A | **P_G + P_T** | **P_ACC** | YELLOW/RED | 006B | **P_G + P_T** | **P_ACC** | YELLOW/RED | YELLOW/RED | YELLOW/RED |
| 007 | M | 007A | **P_T** | **P_ACC** | RED | 007B | **P_T** | **P_ACC** | RED | RED | NA |
| 008 | F | 008A | **P_G + P_T** | **P_ACC + P_GCC** | GREEN/RED | 008B | **P_G + P_T** | **P_ACC + P_GCC** | GREEN/RED | GREEN/RED | NA |
| 009 | F | 009A | **P_G + P_T** | **P_ACC + P_GCC** | GREEN/RED | 009B | **P_G + P_T** | **P_ACC + P_GCC** | GREEN/RED | GREEN/RED | NA |
| 010 | M | 010A | **P_T** | **P_ACC** | RED | 010B | **P_T** | **P_ACC** | RED | RED | NA |
| 011 | F | 011A | **P_G** | **P_ACC + P_GCC** | GREEN/YELLOW | 011B | **P_G** | **P_ACC + P_GCC** | GREEN/YELLOW | GREEN/YELLOW | NA |
| 012 | F | 012A | **P_G + P_T** | **P_ACC + P_GCC** | GREEN/RED | 012B | **P_G + P_T** | **P_ACC + P_GCC** | GREEN/RED | GREEN/RED | NA |
| 013 | M | 013A | **P_G** | **P_GCC** | GREEN | 013B | **P_G** | **P_GCC** | GREEN | GREEN | NA |
| 014 | M | 014A | **P_T** | **P_ACC** | RED | 014B | **P_T** | **P_ACC** | RED | RED | NA |
| 015 | M | 015A | **P_G** | **P_GCC** | GREEN | 015B | **P_G** | **P_GCC** | GREEN | GREEN | NA |
| 016 | M | 016A | **P_T** | **P_ACC** | RED | 016B | **P_T** | **P_ACC** | RED | RED | NA |
| 017 | M | 017A | **P_T** | **P_ACC** | RED | 017B | **P_T** | **P_ACC** | RED | RED | NA |
| 018 | F | 018A | **P_G + P_T** | **P_ACC + P_GCC** | GREEN/RED | 018B | **P_G + P_T** | **P_ACC + P_GCC** | GREEN/RED | GREEN/RED | NA |
| 019 | M | 019A | **P_T** | **P_ACC** | RED | 019B | **P_T** | **P_ACC** | RED | RED | NA |
| 020 | M | 020A | **P_T** | **P_ACC** | RED | 020B | **P_T** | **P_ACC** | RED | RED | NA |
| 021 | M | 021A | **P_G** | **GCC** | GREEN | 021B | **P_G** | **GCC** | GREEN | GREEN | NA |
| 022 | F | 022A | **P_T** | **P_ACC** | RED | 022B | **P_T** | **P_ACC** | RED | RED | NA |
| 023 | M | 023A | **P_G** | **P_GCC** | GREEN | 023B | **P_G** | **P_GCC** | GREEN | GREEN | NA |
| 024 | M | 024A | **P_G** | **P_ACC** | YELLOW | 024B | **P_G** | **P_ACC** | YELLOW | YELLOW | NA |
| 025 | F | 025A | **P_T** | **P_ACC** | RED | 025B | **P_T** | **P_ACC** | RED | RED | NA |
| 026 | M | 026A | **P_T** | **P_ACC** | RED | 026B | **P_T** | **P_ACC** | RED | RED | NA |
| 027 | M | 027A | **P_T** | **P_ACC** | RED | 027B | **P_T** | **P_ACC** | RED | RED | NA |
| 028 | M | 028A | **P_T** | **P_ACC** | RED | 028B | **P_T** | **P_ACC** | RED | RED | NA |
| 029 | F | 029A | **P_T** | **P_ACC** | RED | 029B | **P_T** | **P_ACC** | RED | RED | NA |
| 030 | F | 030A | **P_G + P_T** | **P_ACC** | YELLOW/RED | 030B | **P_G + P_T** | **P_ACC** | YELLOW/RED | YELLOW/RED | NA |
| 031 | M | 031A | **P_T** | **P_ACC** | RED | 031B | **P_T** | **P_ACC** | RED | RED | NA |
| 032 | F | 032A | **P_G** | **P_GCC** | GREEN | 032B | **P_G** | **P_GCC** | GREEN | GREEN | NA |
| 033 | M | 033A | **P_T** | **P_ACC** | RED | 033B | **P_T** | **P_ACC** | RED | RED | NA |
| 034 | M | 034A | **P_T** | **P_ACC** | RED | 034B | **P_T** | **P_ACC** | RED | RED | NA |
| 035 | F | 035A | **P_G + P_T** | **P_ACC** | YELLOW/RED | 035B | **P_G + P_T** | **P_ACC** | YELLOW/RED | YELLOW/RED | NA |
| 036 | F | 036A | **P_G** | **P_ACC** | YELLOW | 036B | **P_G** | **P_ACC** | YELLOW | YELLOW | NA |
| 037 | F | 037A | **P_G** | **P_ACC + P_GCC** | GREEN/YELLOW | 037B | **P_G** | **P_ACC + P_GCC** | GREEN/YELLOW | GREEN/YELLOW | NA |
| 038 | M | 038A | **P_G** | **P_GCC** | GREEN | 038B | **P_G** | **P_GCC** | GREEN | GREEN | NA |
| 039 | M | 039A | **P_T** | **P_ACC** | RED | 039B | **P_T** | **P_ACC** | RED | RED | NA |
| 040 | M | 040A | **P_T** | **P_ACC** | RED | 040B | **P_T** | **P_ACC** | RED | RED | NA |
| 041 | M | 041A | **P_T** | **P_ACC** | RED | 041B | **P_T** | **P_ACC** | RED | RED | NA |
| 042 | M | 042A | **P_G** | **P_GCC** | GREEN | 042B | **P_G** | **P_GCC** | GREEN | GREEN | NA |
| 043 | M | 043A | **P_T** | **P_ACC** | RED | 043B | **P_T** | **P_ACC** | RED | RED | NA |
| 044 | F | 044A | **P_T** | **P_ACC** | RED | 044B | **P_T** | **P_ACC** | RED | RED | NA |
| 045 | F | 045A | **P_G + P_T** | **P_ACC + P_GCC** | GREEN/RED | 045B | **P_G + P_T** | **P_ACC + P_GCC** | GREEN/RED | GREEN/RED | NA |
| 046 | M | 046A | **P_T** | **P_ACC** | RED | 046B | **P_T** | **P_ACC** | RED | RED | NA |
| 047 | F | 047A | **P_T** | **P_ACC** | RED | 047B | **P_T** | **P_ACC** | RED | RED | NA |
| 048 | M | 048A | **P_G** | **P_GCC** | GREEN | 048B | **P_G** | **P_GCC** | GREEN | GREEN | NA |
| 049 | M | 049A | **P_T** | **P_ACC** | RED | 049B | **P_T** | **P_ACC** | RED | RED | NA |
| 050 | F | 050A | **P_T** | **P_ACC** | RED | 050B | **P_T** | **P_ACC** | RED | RED | RED |
| 051 | M | 051A | **P_T** | **P_ACC** | RED | 051B | **P_T** | **P_ACC** | RED | RED | RED |
| 052 | F | 052A | **P_G + P_T** | **P_ACC + P_GCC** | GREEN/RED | 052B | **P_G + P_T** | **P_ACC + P_GCC** | GREEN/RED | GREEN/RED | GREEN/RED |
| 053 | F | 053A | **P_G + P_T** | **P_ACC** | YELLOW/RED | 053B | **P_G + P_T** | **P_ACC** | YELLOW/RED | YELLOW/RED | YELLOW/RED |
| 054 | F | 054A | **P_T** | **P_ACC** | RED | 054B | **P_T** | **P_ACC** | RED | RED | RED |
| 055 | F | 055A | **P_T** | **P_ACC** | RED | 055B | **P_T** | **P_ACC** | RED | RED | RED |
| 056 | F | 056A | **P_G + P_T** | **P_ACC + P_GCC** | GREEN/RED | 056B | **P_G + P_T** | **P_ACC + P_GCC** | GREEN/RED | GREEN/RED | GREEN/RED |
| 057 | F | 057A | **P_T** | **P_ACC** | RED | 057B | **P_T** | **P_ACC** | RED | RED | RED |
| 058 | F | 058A | **P_T** | **P_ACC** | RED | 058B | **P_T** | **P_ACC** | RED | RED | RED |
| 059 | F | 059A | **P_G + P_T** | **P_ACC + P_GCC** | GREEN/RED | 059B | **P_G + P_T** | **P_ACC + P_GCC** | GREEN/RED | GREEN/RED | GREEN/RED |
| 060 | M | 060A | **P_T** | **P_ACC** | RED | 060B | **P_T** | **P_ACC** | RED | RED | RED |
| 061 | M | 061A | **P_T** | **P_ACC** | RED | 061B | **P_T** | **P_ACC** | RED | RED | RED |
| 062 | M | 062A | P_T | P_ACC | RED | 062B | P_T | P_ACC | RED | RED | RED |
| 063 | F | 063A | **P_G** | **P_ACC** | YELLOW | 063B | **P_G** | **P_ACC** | YELLOW | YELLOW | NA |
| 064 | M | 064A | **P_T** | **P_ACC** | RED | 064B | **P_T** | **P_ACC** | RED | RED | NA |
| 065 | M | 065A | **P_G** | **P_ACC** | YELLOW | 065B | **P_G** | **P_ACC** | YELLOW | YELLOW | YELLOW |
| 066 | F | 066A | **P_G + P_T** | **P_ACC + P_GCC** | GREEN/RED | 066B | **P_G + P_T** | **P_ACC + P_GCC** | GREEN/RED | GREEN/RED | GREEN/RED |
| 067 | M | 067A | **P_G** | **P_ACC** | YELLOW | 067B | **P_G** | **P_ACC** | YELLOW | YELLOW | YELLOW |
| 068 | F | 068A | **P_G + P_T** | **P_ACC + P_GCC** | GREEN/RED | 068B | **P_G + P_T** | **P_ACC + P_GCC** | GREEN/RED | GREEN/RED | GREEN/RED |
| 069 | F | 069A | **P_T** | **P_ACC** | RED | 069B | **P_T** | **P_ACC** | RED | RED | RED |
| 070 | F | 070A | **P_G** | **P_ACC** | YELLOW | 070B | **P_G** | **P_ACC** | YELLOW | YELLOW | YELLOW |

APPENDIX 2

### **PROCEDURE PART I – REACTION SET-UP**

1. Thaw all components to room temperature (supermix, cDNA samples, forward and reverse primer, probes).
2. Mix the following:

|  |  |
| --- | --- |
| **Component** | **Volume per 25 ul Reaction** |
| **Supermix for probes** | 12.50 |
| **Forward Primer** | 2.25 |
| **Reverse Primer** | 2.25 |
| **FAM probe/primer** | 1.25 |
| **HEX probe/primer** | 1.25 |
| **cDNA** | 5.50 |
| **Total** | **25.00** |

1. Combine all components from above, except sample (cDNA). Once mixed, vortex briefly (2-5 seconds), then spin down with a centrifuge.
2. In PCR strips, aliquot 19.5ul in each tube. In a blank tube add 5.5 ul of water. In sample tubes at 5.5 ul of cDNA. All tubes should have 25 ul total. Spin down on centrifuge.

### **PROCEDURE PART II – DROPLET GENERATION**

1. Open the DG8 cartridge cassette by simultaneously pushing on the upper and lower sides at the center of the cassette. Place a new DG8 cartridge in the empty cassette and close the assembly by pushing the left and right ends towards each other.
2. Make sure your PCR reactions are mixed well and spun down.
3. Transfer exactly **25 μl** of each **PCR reaction** into the **middle row** of the DG8 cartridge.
4. Pour enough oil into a disposable reservoir and pipette (using multichannel) exactly **70 μl** of droplet generation **oil** **for probes** into the **bottom wells** of the DG8 cartridge.
5. Attach a gasket across the top of the DG8 cartridge.
6. Turn QX200 droplet generator on, open the device by pressing the button on top and gently place the DG8 cartridge with gasket into the droplet generator.
7. Close the lid of the droplet generator by pressing the button on top. The generator will automatically generate the droplets in the top row of the cartridge.

### **PROCEDURE PART III – PCR**

1. Remove the gasket from the DG8 cartridge and pipet the droplets (40 μl) from the top wells of the cartridge into a PCR plate. Pipetting too fast can break the droplets.
2. Turn on the Bio-Rad PX1 PCR plate sealer. Make sure to remove the plate holder before heating.
3. Put a pierceable foil heat seal on top of the PCR plate and heat seal using Bio-Rad’s PX1 PCR plate sealer.
4. Put the PCR plate in the C1000 96 deep-well thermal cycler and start the PCR (settings below).

| **Item** |  | **Temperature (°C)** | **Time** | **Ramp Rate** |
| --- | --- | --- | --- | --- |
| **ENZYME ACTIVATION** | | 95 | 10 min | 1 C/s |
| **DENATURATION** | 48 cycles | 94 | 30 s |  |
| **ANNEALING** |  | 65 | 1 min |  |
|  | | 98 | 10 min |  |
| **HOLD** | | 4 | ∞ |  |
|  | | |  |  |

1. After the PCR has finished, take the plate out of the thermal cycler and check if all the wells have the same volume. You should clearly see 2 separate phases.

### **PROCEDURE PART IV – DROPLET READING**

1. Place the secured plate and plate holder in the droplet reader. Push the button again to close the droplet reader.
2. Confirm the three indicator lights are green before proceeding with the run.
3. Click on **Setup** in the Quantasoft software to define your experiment. Here you can enter information about the samples, assays, and experiments. Select the wells you wish to analyze and indicate the location of the blank.
4. Click **Run** to start the droplet reading. The far right indicator light should flash green.
5. When the droplet reading is fully completed, all four indicator lights will be solid green. You can now open the door, remove the plate holder and discard your PCR plate (or save it for further applications).
6. Analyze your results:
   1. Review the 2D plots generated by the software for each well, which display fluorescence amplitudes for FAM and HEX channels.
   2. Verify or set the thresholds for positive and negative droplets based on control samples:
      1. Amplitudes around 1000 are considered negative;
      2. Amplitudes between 3000 and 5000 are considered positive.
   3. Adjust thresholds as needed, ensuring alignment with positive control and non-template control results.
   4. Confirm expected patterns in control wells:
      1. Positive control wells should show distinct populations of positive droplets in both FAM and HEX channels.
      2. Non-template control wells should only display negative droplets, confirming the absence of contamination.
   5. Assign each sample to its corresponding target SNPs based on channel fluorescence.
